# 812 Evaluation of Dermal and Epidermal Replacement Strategies for the Treatment of Full-thickness Wounds

**DOI:** 10.1093/jbcr/irac012.360

**Published:** 2022-03-23

**Authors:** William L Hickerson, Lauren Mangum, Cameron C Bell, Katie A Bush

**Affiliations:** Spectral MD, Avita Medical, AccessPro Med, Memphis, Tennessee; AVITA Medical, Fishers, Indiana; AVITA Medical, Denver, Colorado; AVITA Medical, Marblehead, Massachusetts

## Abstract

**Introduction:**

In full-thickness (FT) wounds, autologous skin cell suspension (ASCS) is used in combination with widely meshed split-thickness skin grafts (mSTSGs) to obtain definitive closure, reducing donor skin requirements compared to traditional autografting (AG) techniques. In the treatment of these wounds, dermal matrices (DMs) are also often utilized to address various challenges including need for temporization, mitigation of contour defects, covering avascular structures, and modulation of scar formation. Varying DMs exist and are composed of different biologic and synthetic materials designed to address these clinical needs. The purpose of this study was to compare outcomes obtained in FT wounds using 3 different DMs coupled with ASCS+mSTSG in immediate or delayed AG procedures.

**Methods:**

A FT excisional porcine wound model was used. DMs evaluated included a single-layer dermal matrix composed of bovine dermal collagen and elastin (Col/E), a bilayer construct composed of a bovine collagen-glycosaminoglycan with a silicone epidermal layer (Col/GAG), and a synthetic bilayer polyurethane DM (Poly/U).

DMs were applied, managed, and grafted following manufacturer’s recommendations. AG included ASCS+mSTSG (1:80 expansion, 3:1 mesh). Wounds were evaluated for inflammation, infection, DM take, AG take, re-epithelialization, and contracture over 49 days post-excision. Additionally, biopsies were evaluated to further inform tissue generation and healing outcomes.

**Results:**

Results related to healing outcomes are reported in the table below. Inflammation at wound margins was noted during acute phase of healing for all DMs and visual cues for infection were noted in 1 wound for Col/E, 3 wounds for Col/GAG causing partial loss of DM, and 1 wound for Poly/U. All signs of infection were resolved by day 14.

**Conclusions:**

ASCS+mSTSG can be used successfully over DMs composed of various materials in both immediate and delayed AG procedures. No difference was observed on percent AG take between the DMs, however the data suggest that time to definitive closure is impacted based on utilized DM and AG strategy, with potential implications on contracture.

